# Twelve-month follow-up of a controlled trial of a brief behavioural intervention to reduce psychological distress in young adolescent Syrian refugees

**DOI:** 10.1017/S2045796024000817

**Published:** 2024-12-23

**Authors:** Richard A. Bryant, Rand Habashneh, Maha Ghatasheh, Aiysha Malik, Ibrahim Said Aqel, Katie S. Dawson, Sarah Watts, Mark J. D. Jordans, Felicity L. Brown, Mark van Ommeren, Aemal Akhtar

**Affiliations:** 1 School of Psychology, University of New South Wales, Sydney, Australia; 2 Brain Dynamics Centre, Westmead Institute for Medical Research, Sydney, Australia; 3 King Hussein Foundation, Institute for Family Health, Amman, AMM, Jordan; 4 Department of Mental Health and Substance Abuse, World Health Organization, Geneva, Switzerland; 5 Research and Development Department, War Child Alliance, Amsterdam, the Netherlands; 6 Amsterdam Institute of Social Science Research, University of Amsterdam, Amsterdam, the Netherlands; 7 Department of Clinical, Neuro and Developmental Psychology, World Health Organization Collaborating Centre for Research and Dissemination of Psychological Interventions, Vrije Universiteit, Amsterdam, the Netherlands

**Keywords:** adolescents, controlled trial, internalising, psychological treatment, refugees

## Abstract

**Aims:**

The majority of studies of mental health interventions for young adolescents have only evaluated short-term benefits. This study evaluated the longer-term effectiveness of a non-specialist delivered group-based intervention (Early Adolescent Skills for Emotions; EASE) to improve young adolescents’ mental health.

**Methods:**

In this single-blind, parallel, controlled trial, Syrian refugees aged 10-14 years in Jordan who screened positive for psychological distress were randomised to receive either EASE or enhanced usual care (EUC). Primary outcomes were scores on the Paediatric Symptom Checklist (PSC) assessed at Week 0, 8-weeks, 3-months, and 12 months after treatment. Secondary outcomes were disability, posttraumatic stress, school belongingness, wellbeing, and caregivers’ reports of distress, parenting behaviour, and their perceived children’s mental health.

**Results:**

Between June, 2019 and January, 2020, 185 adolescents were assigned to EASE and 286 to EUC, and 149 (80.5%) and 225 (78.7%) were retained at 12 months, respectively. At 12 months there were no significant differences between treatment conditions, except that EASE was associated with less reduction in depression (estimated mean difference -1.6, 95% CI –3.2 to -0.1; p=.03; effect size, -0.3), and a greater sense of school belonging (estimated mean difference -0.3, 95% CI –5.7 to -0.2; p=.03; effect size, 5.0).

**Conclusions:**

Although EASE led to significant reductions in internalising problems, caregiver distress, and harsh disciplinary parenting at 3-months, these improvements were not maintained at 12 months relative to EUC. Scalable psychological interventions for young adolescents need to consider their ongoing mental health needs. Prospectively registered: ACTRN12619000341123.

## Introduction

Children and adolescents in low- and middle-income countries (LMIC) are disproportionately exposed to war, disasters, overcrowding, poverty, and humanitarian crises. These factors can contribute to the observed higher rates of common mental disorders in adolescents in these countries (Blackmore *et al.*, 2020). Despite the significant need for mental health services in these settings, there is typically a scarcity of mental health specialists available to provide mental health treatment (Patel *et al.*, 2018). This situation has led to a shift towards task-shifting approaches in which trained non-specialists deliver mental health programmes. Although this initiative has been shown to be moderately effective in meta-analyses (Singla *et al.*, 2017), recent evidence indicates that we are lacking evidence for programmes that effectively reduce common mental health problems, such as anxiety and depression, in adolescents (Barbui *et al.*, 2020; Purgato *et al.*, 2018).

In response to this knowledge gap, the World Health Organization (WHO) developed the early adolescents skills for emotions (EASE) programme designed to reduce internalising problems, such as anxiety and depression, in young adolescents. This programme was a developmentally appropriate adaptation of the WHO’s Problem Management Plus (Dawson *et al.*, 2015), which is a lay-provider delivered five-session programme that has been shown to have good effects in reducing psychological distress (Bryant *et al.*, 2017; Jordans *et al.*, 2021; Rahman *et al.*, 2016). The EASE programme comprises seven group sessions in which adolescents are instructed in psychoeducation, arousal reduction, problem management, behavioural activation, and accessing social supports, as well as three group sessions for caregivers that reinforce strategies taught to the adolescents and briefly promote positive parenting (Dawson *et al.*, 2019). The first controlled trial of EASE was conducted in a large sample of young adolescent Syrian refugees in Jordan where participants were randomised to receiving either EASE or enhanced usual care (EUC) (Bryant *et al.*, 2022). This study found that EASE resulted in greater reduction in internalising symptoms in the adolescents, as well as less psychological distress in the caregivers.

Despite the initial positive reports regarding the efficacy of EASE, the primary study focused on short-term outcomes by reporting 3-month follow-up data. The absence of longer-term follow-up of the effects of EASE is problematic because young adolescents in LMIC, and particularly those affected by conflict or humanitarian crisis, typically experience ongoing stressors that can contribute to poorer mental health (Miller and Rasmussen, 2017). This is especially relevant to young adolescents for whom developmental and hormonal changes often interact with environmental factors to impact mental health (Sisk and Gee, 2022). Accordingly, it is important to determine whether the skills taught in the EASE programme lead to benefits over the long-term. It is noteworthy that whereas adult refugees who Problem Management Plus reported less psychological distress at a 3-month assessment relative to EUC (Bryant *et al.*, 2022a), this benefit was not evidenced at 12-month follow-up (Bryant *et al.*, 2022b). To address the issue of the longer-term effects of a brief lay-delivered psychological intervention for adolescents in a LMIC, this study reports a 12-month follow-up of the previous trial of EASE conducted in Jordan (Bryant *et al.*, 2022).

## Method

### Study design

This two-arm, single-blind RCT was conducted in Amman, Jordan. The study was conducted in collaboration with the Institute for Family Health, a national non-governmental agency in Jordan, where there are an estimated 1.4 million Syrian refugees. The project was prospectively registered (Australian and New Zealand Clinical Trials Registry, no. ACTRN12619000341123), and was approved locally by the Ethics Committee of Al Basheer Hospital in Jordan, the University of New South Wales Human Research Ethics Committee, and the WHO Ethical Review Committee. The trial protocol is available in supplementary information (S1 Text). This study is reported as per the Consolidated Standards of Reporting Trials statement.

### Participants

Participants were enlisted in the trial if they met the following inclusion criteria: (a) Syrian refugee; (b) aged 10–14 years; (c) resided with a related caregiver who could provide legal consent; and (d) scored ≥15 on the Paediatric Symptom Scale (PSC-17) (Gardner *et al.*, 1999). The PSC-17 is a 17-item questionnaire that assesses psychological distress in children, with a range of 0 –34; a cut-off ≥15 has been shown to indicate psychological distress. Exclusion criteria were as follows: (a) unaccompanied minor; (b) minors with an unrelated caregiver and (c) significant developmental, cognitive or neurological impairments as determined by four items from an adapted version of the Ten Questions instrument (Durkin *et al.*, 1995); or (d) imminent risk of suicide. Participants were identified following door-to-door visits in Amman and inviting Syrian refugee adolescents and their caregivers to participate. Informed consent was obtained from caregivers and assent from the adolescents in two stages to participate in (a) the screening and (b) the EASE trial; participation required written informed consent, except oral consent was accepted for illiterate participants.

Eligible adolescents were randomised to either EASE or EUC (on a 1:1.6 ratio) by staff at the University of New South Wales (Australia) who were independent of the trial using computer-generated random number sequences. EASE comprised 7 weekly 1.5-hour group sessions for adolescents (8–10 people per group and groups were gender specific). As described elsewhere (Dawson *et al.*, 2019), EASE comprised psychoeducation about stress, and provided strategies on how to identify emotions, reducing arousal, behavioural activation, problem solving strategies, seeking social support, and relapse prevention. A caregiver of each adolescent was invited to three 2-hour group sessions (8–10 people per group) at 2 weekly intervals concurrently with the adolescent sessions. The caregiver sessions informed caregivers about the skills being taught to the adolescents, as well as psychoeducation and skills for them to further help their child cope with stress, brief skills in positive parenting skills, and strategies to manage the caregivers’ stress. Groups were led by two facilitators who had no specialist mental health qualifications but received 8 days of training on the EASE protocol, as well as group facilitation skills. Facilitators received weekly supervision through the trial by a Jordanian supervisor (MG) with >15 years in psychosocial programmes and who participated in a training of trainer course on the EASE intervention.

EUC comprised a single 30-minute family session conducted in the participant’s home by a community health worker. In this visit feedback was given about the adolescent’s assessment responses, instructions on simple coping strategies, and a list of local psychosocial services that could provide further support.

### Outcomes

Assessments were conducted by Arabic speaking Jordanian assessors, who received three days of training. To address poor literacy, even though the assessment measures were self-report scales, the questions were verbally administered by assessors and then entered participants’ responses on tablets. Assessors were blind to treatment allocation, and at each assessment assessors were instructed to guess which treatment arm the person was assigned to.

### Primary outcome

The primary outcome measure was the adolescents’ self-reported responses on the PSC-35, a 35-item instrument scored on a 3-point Likert scale (0 = *never*, 2 = *often*) (Gardner *et al.*, 1999). The primary subscales index internalising, externalising, and attentional problems, as well as providing a total score of children’s mental health, with higher total scores reflecting more severe psychosocial problems. Importantly, the PSC has been validated in Middle Eastern settings (Monir *et al.*, 2016).

### Secondary outcomes

The Patient Health Questionnaire, adolescent version (PHQ-A) assessed symptoms of depression (Johnson *et al.*, 2002). The PHQ-A is a 9-item symptom checklist corresponding to symptoms of depression experienced in the past week, with a score range of 0–36 and higher total scores reflecting more severe symptoms of depression; this scale has been validated in refugees in Jordan (Al-Amer *et al.*, 2020). Post-traumatic stress was assessed with the Children’s Revised Impact of Events Scale (CRIES-13) (Child and War Foundation, 2005), which measures intrusive memories, avoidance and arousal, with a score range of 0–65 and higher total scores reflecting more severe symptoms of post-traumatic stress; the CRIES-13 has been validated in Middle Eastern youth (Veronese et al. 2022). Wellbeing was assessed using the self-reported Warwick Edinburgh Mental Wellbeing Scale (WEMWBS) (Tennant *et al.*, 2007), with a score range of 0–50 and higher scores indicating greater wellbeing. Adolescents’ sense of belonging and psychological engagement in school was measured through the Psychological Sense of School Membership (PSSM), with a score range of 0–90 and higher scores indicating a greater sense of belonging (Goodenow, 1993). Daily functioning was indexed with a scale developed for the EASE trial in which adolescents rated nine items representing their daily activities, with higher scores reflecting greater impairment.

Caregivers were administered the caregiver version of the PSC-35 to assess their perceptions of their child’s psychological distress. Caregivers’ distress was assessed using the Kessler Distress Scale (K6), with a total score range of 6–30 and higher scores indicating greater distress (Kessler *et al.*, 2002); the K6 has been validated with Arabic refugees (Segal *et al.*, 2018). Parenting style was assessed with the Alabama Parenting Questionnaire (APQ), which measures (a) parental involvement, (b) poor supervision and monitoring, (c) positive parenting, (d) inconsistent discipline and (e) corporal punishment (Maguin *et al.*, 2016), with higher scores indicating greater strength of the relevant subscale; an Arabic version of the APQ has been validated (Badahdah and Le, 2016). Adolescents’ exposure to potentially traumatic events was measured by caregivers’ reports on a 26-item traumatic events checklist.

### Statistical analyses

The sample size was determined to require 470 participants, with a project attrition rate of 33% at the 3-month primary outcome timepoint. Details of the power analysis are reported in the trial protocol (Brown *et al.*, 2019). An allocation ratio of EASE to EUC arms of 1:1.6 accommodated the effects of groups involved in EASE relative to the individual EUC. No sample size calculation was conducted for the 12-month follow-up because it was a secondary analysis.

The major analyses focused on intention-to-treat. Linear mixed models were used to compute the differential effects of the treatment arms. Fixed (intervention, time of assessment) effects and their interactions were included in the unstructured models to provide an index of the relative effects of the treatments; time of assessment included baseline, post-treatment, 3-month, and 12-month follow-up. Fixed effects parameters were tested with the Wald test (t-test, *p* < .05, two-sided) and 95% confidence intervals. Missing data were assumed to be random on the basis that participants completing the 12-month assessment and those who were missing did not differ in terms of any demographic or outcome measures at baseline. We also conducted a completer analysis, including only participants who completed the 12-month follow-up.

## Results

**Figure 1.**
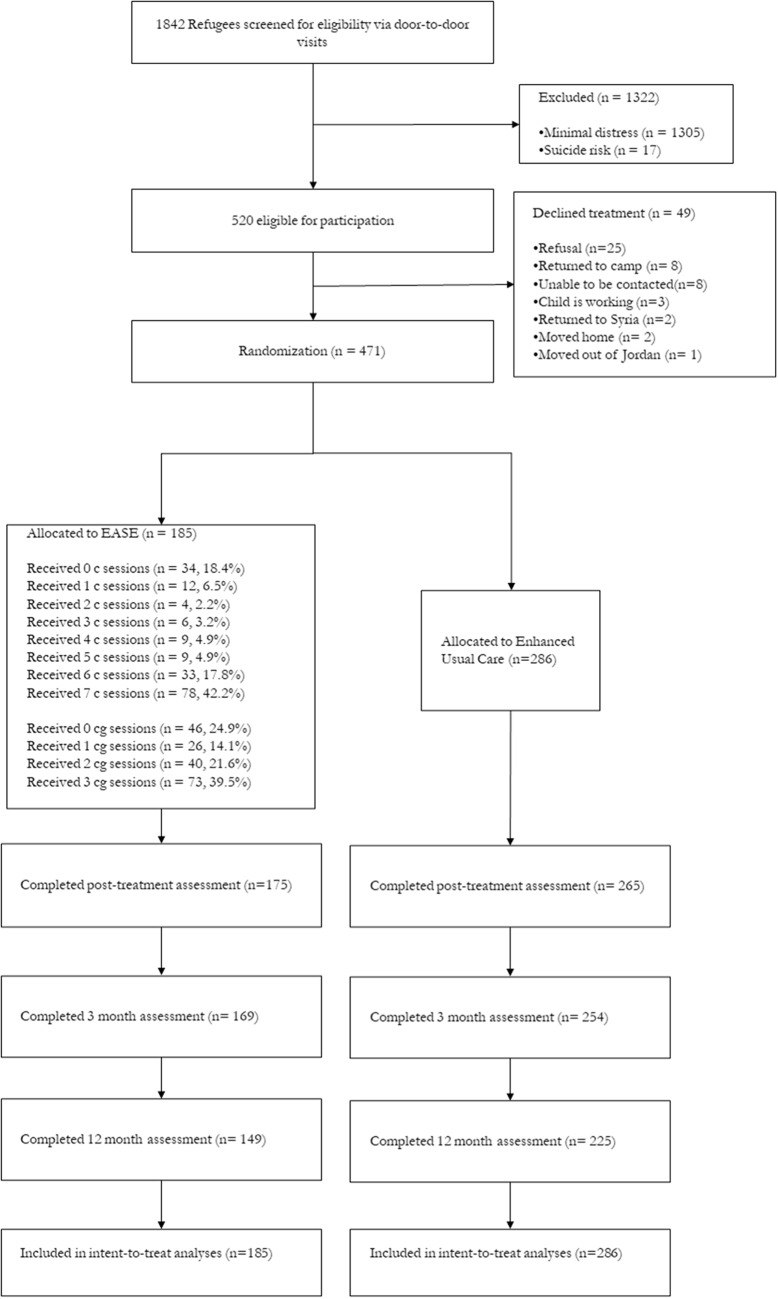
CONSORT flow diagram of progress through phases of a randomized trial comparing the early adolescent skills for emotions (EASE) intervention vs enhanced usual care (EUC) in young adolescent Syrian refugees, Jordan.

Participants were enrolled between June, 2019, and January, 2020, and the final 12-month assessments were completed by August, 2021; in this context it is worth noting that baseline assessments were conducted prior to the COVID-19 pandemic, whilst EASE sessions and subsequent assessments occurred after the pandemic had widely affected Jordan. There were 471 randomised (185 into EASE and 286 into EUC). The 12-month assessment was conducted for 374 participants (79.4%) participants, with comparable proportions of participants retained in the EASE condition (149, 80.5%) and EUC (225, 78.7%) conditions, χ^2^ = 0.2, *p* = .60. Participants who were lost at follow-up did not differ from those who were retained in terms of gender, time since leaving Syria, trauma exposure or baseline scores on any outcome measures (see Table 1). Participants who were retained were younger than those lost to follow-up, however there was only 3 months difference between these two groups. The flowchart of participant recruitment and retention is reported in Fig. 1. Details of the participants are reported in full in the prior report of the study (Bryant *et al.*, 2022) and reported in online supplementary Tables S2 and S3. The mean age of participants was 11.6 years (SD 1.3), equally distributed across gender (49.5% females) and most participants had left their home in Syria at least 7 years ago (73.9%). At baseline, adolescents had been exposed to an average of 6.89 (SD 3.83) traumatic events, with the most common events having had lived in a war zone (60.7%), experiencing danger during flight from Syria (89.0%), seeing dead bodies (71.3%), serious injury to friends or family (67.1%) and lack of food or water (79.2%). No adverse events were attributable to the interventions or the trial. Assessors correctly guessed the participant’s assigned condition at chance rates at both the post-treatment (49.0%), follow-up (56.5%) and 12-month (51.4%) assessments.

**Table 1.** Participant characteristics for participants retained and not retained at 12-month assessment

	Retained (*n* = 374)	Not retained (*n* = 97)	*T/χ^2^*	*P*
Adolescent variables				
Female, *n* (%)	187 (50.0%)	46 (47.4%)	0.2	.65
Age, *y*	11.6 ± 1.3	11.9 ± 1.3		
Time since leaving Syria, *y*	2.7 ± 0.5	2.8 ± 0.4	−1.6	.12
Number of traumatic events, *M*	6.8 ± 3.7	7.1 ± 4.5	−0.8	0.42
Education, *n* (%)			2.8	0.43
No school	10 (2.7%)	1 (1.0%)		
Primary school	266 (71.1%)	202 (70.6%)		
Middle school	94 (25.1%)	30 (30.9%)		
High school	4 (1.1%)	2 (2.1%)		
Adolescent reports				
PSC internalising	5.4 ± 1.9	5.5 ± 1.9	−0.7	0.51
PSC externalising	7.3 ± 2.5	7.4 ± 2.7	−0.7	0.49
PSC attention	5.5 ± 2.0	5.5 ± 2.0	−0.1	0.97
PSC total	32.7 ± 8.8	31.5 ± 8.3	1.1	0.2
PHQ9-A	15.3 ± 5.8	15.2 ± 6.4	0.1	0.94
CRIES	23.8 ± 12.2	22.9 ± 11.4	0.7	0.49
WEBWBS	39.7 ± 9.5	41.3 ± 8.9	−1.4	0.16
Functioning	16.9 ± 7.3	16.0 ± 7.1	1.3	0.21
PSM	2.8 ± 0.7	2.9 ± 0.7	−1.6	0.11
Caregiver reports				
PSC internalising	4.5 ± 2.4	4.2 ± 2.4	1.3	0.2
PSC externalising	5.2 ± 2.7	4.8 ± 2.7	1.1	0.26
PSC attention	5.6 ± 2.4	5.4 ± 2.3	0.8	0.4
PSC total	27.6 ± 10.9	26.4 ± 13.2	0.8	0.41
Alabama involvement	29.6 ± 6.0	30.6 ± 6.2	−1.4	0.15
Alabama positive parenting	19.6 ± 3.7	20.2 ± 3.8	−1.4	0.15
Alabama supervision	17.5 ± 6.3	17.2 ± 6.7	0.5	0.65
Alabama discipline	15.4 ± 3.6	15.1 ± 3.7	0.7	0.51
Alabama punishment	6.6 ± 2.3	6.5 ± 2.6	0.5	0.62
K6	14.6 ± 5.2	15.7 ± 5.5	−1.9	0.06

*Note:* PSC = Paediatric Symptom Checklist; PHQ-9A = Patient Health Questionnaire Adolescent Version. CRIES = Children’s Revised Impact of Events Scale. WEBWBS = Warwick Edinburgh Mental Wellbeing Scale. PSM = Psychological Sense of School Membership. K6 = Kessler Psychological Distress Scale. Alabama = Alabama Parenting Questionnaire. Continuous measures reported as means and standard deviations (±).

The primary and secondary outcomes at each timepoint are presented in Table 2. At the 12-month follow-up assessment, there were no significant differences between participants who received EASE and EUC in terms of scores on the PSC-internalising, (estimated mean difference 0.5, 95% CI 0.0–1.0; *p* = .08; effect size, 0.3), externalising (estimated mean difference −0.2, 95% CI −0.8 to 0.4; *p* = .51; effect size, -0.1), and attention subscales (estimated mean difference 0.1, 95% CI −2.4 to 0.6; *p* = .74; effect size, 0.1), or PSC total scores (estimated mean difference 0.1, 95% CI −2.4 to 2.6; *p* = .92; effect size, 0.0) scores. Notably, there were significant reductions at 12 months in both conditions relative to baseline in terms of internalising (estimated mean difference 2.3, 95% CI–2.0 to 0.6; *p* < .001), externalising (estimated mean difference 4.9, 95% CI 4.6– 5.2; *p* < .001), attention (estimated mean difference 2.3, 95% CI 2.0–2.5; *p* < .001), and total scores (estimated mean difference 17.2, 95% CI −15.9 to 18.4; *p* < .001) scores.

**Table 2.** Summary statistics and results from mixed model analysis of primary and secondary outcomes

		Descriptive statistics				
		EASE (*n* = 168)	EUC (*n* = 189)	Mixed model analysis		
Primary and secondary outcomes	Visit	Estimated mean (SE)	Estimated mean (SE)	Difference in LS mean (95%CI)	P-value	Effect size
Adolescent reported outcomes						
PSC internalising	Baseline	5.6 (0.1)	5.3 (0.1)			
	6 weeks	2.9 (0.1)	3.3 (0.1)	0.7 (0.2, 1.2)	0.003	0.4
	3 months	3.0 (0.1)	3.4 (0.1)	0.7 (0.2, 1.2)	0.005	0.4
	12 months	3.2 (0.2)	3.3 (0.1)	0.5 (0.0, 1.0)	0.08	0.3
PSC externalising	Baseline	7.2 (0.2)	7.4 (0.1)			
	6 weeks	4.4 (0.2)	4.6 (0.1)	0.1 (−0.5, 0.7)	0.72	0
	3 months	4.5 (0.2)	4.4 (0.1)	−0.2 (−0.8, 0.4)	0.43	−0.1
	12 months	2.4 (0.2)	2.3 (0.1)	−0.2 (−0.8, 0.4)	0.51	−0.1
PSC attention	Baseline	5.6 (0.1)	5.6 (0.1)			
	6 weeks	3.0 (0.1)	3.3 (0.1)	0.4 (−0.1, 0.8)	0.15	0.2
	3 months	3.2 (0.1)	3.2 (0.1)	0.0 (−0.5, 0.5)	0.97	0.2
	12 months	8.2 (0.1)	8.4 (0.1)	0.1 (−0.4, 0.6)	0.74	0.1
PSC total	Baseline	32.4 (0.5)	32.5 (0.5)			
	6 weeks	18.4 (0.7)	20.1 (0.6)	1.8 (−0.6, 4.2)	0.14	0.2
	3 months	18.9 (0.7)	18.8 (0.6)	0.1 (−2.4, 2.5)	0.96	0
	12 months	50.2 (0.7)	15.2 (0.6)	0.1 (−2.4, 2.6)	0.92	0
CRIES	Baseline	24.2 (0.7)	23.3 (0.6)			
	6 weeks	18.4 (0.7)	18.3 (0.6)	0.7 (−2.5, 3.4)	0.77	0.1
	3 months	18.8 (0.8)	18.9 (0.6)	0.8 (−2.0, 3.6)	0.56	0.1
	12 months	24.4 (0.8)	21.6 (0.7)	−1.9 (−4.8, 1.0)	0.2	−0.2
PHQ-A	Baseline	15.1 (0.4)	15.4 (0.3)			
	6 weeks	12.8 (0.4)	12.4 (0.3)	−0.7 (−2.2, 0.7)	0.32	−0.1
	3 months	12.4 (0.4)	12.3 (0.3)	−0.4 (−1.9, 1.1)	0.61	−0.1
	12 months	11.2 (0.4)	9.9 (0.4)	−1.6 (−3.2, −0.1)	0.03	−0.3
Functioning	Baseline	16.7 (0.5)	16.8 (0.4)			
	6 weeks	13.3 (0.5)	13.7 (0.4)	0.4 (−1.3, 2.0)	0.67	0.1
	3 months	14.5 (0.5)	14.4 (.4)	−0.2 (−1.8, 2.0)	0.85	0
	12 months	11.4 (0.5)	11.0 (0.4)	−0.4 (−2.1, 1.2)	0.61	−0.1
WEBWBS	Baseline	40.7 (0.6)	39.6 (0.4)			
	6 weeks	45.1 (0.6)	45.0 (0.5)	0.9 (−1.0, 2.9)	0.33	0.1
	3 months	44.9 (0.6)	45.1 (0.5)	1.4 (−0.7, 3.4)	0.19	−0.3
	12 months	49.8 (0.6)	48.6 (0.5)	−0.1 (−2.2, 2.0)	0.92	0
PSSM	Baseline	50.1 (.8)	52.2 (0.6)			
	6 weeks	53.3 (.8)	53.9 (0.7)	−1.6 (−4.3, 1.2)	0.26	−2.7
	3 months	54.3 (.8)	53.8 (0.7)	−2.6 (−5.42, 0.20)	0.07	−4.3
	12 months	52.2 (0.8)	51.3 (0.63)	−3.0 (−5.7, −0.2)	0.03	−5
Caregiver reported outcomes						
K6	Baseline	15.4 (0.3)	14.5 (0.3)			
	6 weeks	16.9 (0.4)	16.9 (0.3)	0.9 (−0.3, 2.2)	0.13	0.2
	3 months	16.8 (0.4)	17.7 (0.3)	1.9 (0.7, 3.1)	0.001	0.4
	12 months	16.1 (0.4)	16.2 (0.3)	1.1 (−0.1, 2.3)	0.08	0.2
Alabama involvement	Baseline	29.6 (0.4)	29.9 (0.3)			
	6 weeks	30.6 (0.4)	30.7 (0.3)	−0.2 (−1.6, 1.2)	0.81	0
	3 months	30.7 (0.4)	30.5 (0.3)	−0.5 (−1.9, 0.9)	0.49	0
	12 months	33.5 (0.4)	32.6 (0.4)	−1.1 (−2.5, 0.3)	0.13	−0.2
Alabama supervision	Baseline	17.4 (0.5)	17.5 (0.4)			
	6 weeks	17.4 (0.5)	16.5 (0.4)	−1.0 (−2.7, 0.6)	0.22	−0.3
	3 months	16.8 (0.5)	15.7 (0.4)	−1.2 (−2.8, 0.5)	0.17	−0.2
	12 months	19.3 (0.5)	18.1 (0.4)	−1.4 (−3.1, 0.4)	0.12	−0.4
Alabama positive parenting	Baseline	19.7 (0.3)	19.7 (0.2)			
	6 weeks	19.8 (0.3)	20.0 (0.2)	0.2 (−0.7, 1.1)	0.67	.0.1
	3 months	20.0 (0.3)	20.0 (0.2)	0.0 (−0.9, 0.9)	0.95	0
	12 months	22.0 (0.3)	21.4 (0.3)	−0.5 (−1.4, 0.4)	0.29	−0.2
Alabama discipline	Baseline	15.5 (0.3)	15.2 (0.2)			
	6 weeks	13.7 (0.3)	13.9 (0.2)	0.5 (−0.4, 1.4)	0.25	0.1
	3 months	13.3 (0.3)	14.2 (0.2)	1.2 (0.3, 2.2)	0.009	0.3
	12 months	16.4 (0.2)	16.4 (0.2)	−0.6 (−1.5, 0.4)	0.26	0.5
Alabama punishment	Baseline	6.6 (0.2)	6.6 (0.1)			
	6 weeks	6.2 (0.2)	5.7 (0.1)	−0.4 (−0.9, 0.1)	0.13	−0.2
	3 months	5.7 (0.2)	5.7 (0.1)	0.1 (−0.5, 0.6)	0.85	−0.3
	12 months	5.8 (0.2)	5.9 (0.1)	0.2 (−0.4, 0.7)	0.52	0
PSC total score	Baseline	32.6 (0.7)	32.5 (0.5)			
	6 weeks	18.4 (0.7)	20.1 (0.6)	1.8 (−0.6, 4.2)	0.14	0.2
	3 months	18.9 (0.7)	18.8 (0.6)	0.1 (−2.4, 2.5)	0.96	−0.1
	12 months	15.3 (0.7)	15.4 (0.6)	0.1 (−2.3, 2.6)	0.91	0
PSC Attention	Baseline	5.6 (0.2)	5.5 (0.1)			
	6 weeks	3.7 (0.2)	4.0 (0.1)	0.3 (−0.2, 0.8)	0.25	0.1
	3 months	3.9 (0.2)	4.0 (0.1)	0.1 (−0.5, 0.6)	0.77	0
	12 months	3.2 (0.2)	3.2 (0.1)	0.0 (−0.6, 0.6)	0.94	0
PSC internalising	Baseline	4.4 (0.2)	4.5 (0.1)			
	6 weeks	2.8 (0.2)	3.0 (0.1)	0.1 (−0.5, 0.7)	0.73	0
	3 months	2.7 (0.2)	2.7 (0.1)	−0.2 (−0.7, 0.4)	0.6	−0.1
	12 months	3.0 (.2)	3.5 (0.1)	0.3 (−0.3, 0.9)	0.28	0.1
PSC externalising	Baseline	5.4 (0.2)	4.9 (0.1)			
	6 weeks	3.5 (0.2)	3.9 (0.1)	0.9 (−0.2, 1.1)	0.003	0.3
	3 months	3.8 (0.2)	3.7 (0.1)	0.4 (−0.2, 1.1)	0.16	0.1
	12 months	2.6 (0.2)	2.5 (0.2)	0.4 (−0.3, 1.0)	0.24	0.1

In terms of secondary outcomes, at 12 months adolescents in the EASE condition reported less reduction in depression (estimated mean difference −1.6, 95% CI −3.2 to −0.1; *p* = .03; effect size, −0.3), and a greater sense of school belonging (estimated mean difference −0.3, 95% CI −5.7 to −0.2; *p* = .03; effect size, 5.0) than those in EUC. There were no other significant differences between conditions on other secondary outcome measures for adolescents or caregivers.

The complete case analysis focusing only on participants retained at the 12-month follow-up did not change the pattern of results observed in the linear mixed models analyses, with the exception that caregivers in the EASE condition had lower K6 scores at 12 months than those in EUC (see Supplement Table S3).

## Discussion

The major finding of this study was that the initial relative benefits in reducing internalising problems among adolescents receiving EASE compared to EUC was no longer significant at 12-months follow-up. We note that there was a trend for EASE to have a persistent benefit (*p* = .08), however this difference was not significant which suggests that the initial relative benefit of EASE compared to EUC weakened over time. It is worth noting that EASE was developed primarily to mitigate internalising problems (Dawson *et al.*, 2019), and so it notable that this was the category of adolescents’ problems that showed a trend towards persistent benefit over 12 months. The relative improvement in caregivers in EASE on psychological distress (on the K6) was also only observed at a marginal level at 12 months (*p* = .08). Further, the relative improvement in inconsistent disciplinary behaviour that was significant at 3 months in caregivers in EASE was no longer apparent at 12 months.

There are several explanations for this pattern of findings. First, the trial was powered to detect a significant effect at the 3-month assessment, and so we recognise that with attrition at 12 months the current analyses were underpowered. However, retention at 12 months was good and exceeded the numbers projected in each treatment arm to detect differences at 3 months. Second, the initial benefits of EASE that were evident at 3 months did not persist to the same extent at 12 months because strategies learnt during the sessions may have not been rehearsed or used in the longer-term, and this contributed to reduced difference between the two treatments. Third, the 3-month follow-up was conducted at a time in Jordan when COVID-19 restrictions were at their peak, and so it is possible that environmental stressors at this time heightened differences between the two treatment arms because those receiving EASE had learnt strategies to manage the stress. In contrast, the 12-month follow-up was conducted at a time when the pandemic restrictions in Jordan had eased to an extent. This interpretation accords with the finding that both conditions were characterised by marked reduction in psychopathology levels at 12 months relative to baseline. Fourth, it is possible that at 12-months there was a regression to the mean for all participants, and this minimised the difference between EASE and EUC.

It is curious that participants in the EASE reported less reduction in depression at 12 months compared to those in EUC. Observation of the estimated means suggests that whereas participants in EUC reported a greater reduction of depression from 3–12 months, depression levels tended to remain more stable for the EASE participants. This may be a spurious finding, partly associated with the restricted sample that was followed up at 12 months. Alternately, it is possible that the lack of maintenance of reduced internalising symptoms in EASE participants, which includes emotional difficulties such as anxiety and depression, is reflected in less reduction of depression at 12 months.

These findings have implications for transdiagnostic scalable interventions for young adolescents. Most evidence-based scalable interventions implemented in LMICs, including the suite of WHO interventions, are time-limited programmes that typically comprise between five and seven sessions. Considering the level of psychological distress, and often experiences of adversity, that characterise people with common mental disorders in LMICs that are exposed to humanitarian crises, it may be overly optimistic to expect brief interventions to have long-lasting benefits, without any follow-up or booster sessions. This result accords with another trial of a scalable intervention with adult Syrian refugees that also found that initial benefits observed at 3 months were not maintained at 12 months (Bryant *et al.*, 2022b). This interpretation is particularly pertinent in the context of the common daily stressors experienced by many people in LMICs, exposed to humanitarian crises. There are several options worthy of further investigation to promote maintenance of better mental health after EASE. First, providing booster sessions or some other form of maintaining EASE strategies may be beneficial for people living with ongoing distress. Second, referral systems to address other psychosocial needs (e.g., poverty, housing needs) may reduce ongoing stressors. Third, stepped care frameworks that triage people with more severe needs (e.g., PTSD) to more targeted interventions or provide specialist care if they not respond optimally to EASE. Fourth, refinement of EASE so it provides more long-lasting benefits. Fifth, it is possible that the limited training and supervision that the non-specialists received may have resulted in attenuated impacts of EASE; more intensive training and/or supervision could be tested in future studies to determine the long-term impact of EASE. These options require careful evaluation of their efficacy and cost-effectiveness to determine the utility of such strategies to maintain initial gains in the context of LMICs affected by humanitarian crises.

Confidence in this study’s results is indicated by a number of study strengths, including long-term follow-up, high retention rate, adherence to the treatment protocols, verified blind assessments, and extensive cultural adaptation of EASE for Syrian refugees. There were also study limitations. We note that the WEMWBS and PSSM have not been validated with young adolescents in Arabic settings, and so caution should be used in interpreting these measures. Also, the two treatment conditions were not matched for weekly contact or group format, and most caregivers were female (predominantly mothers). We also recognise that although retention at 12 months was reasonable (79.4%) and there were no baseline differences between those who did and did not complete the 12-month assessment, it is possible that those who were not retained at 12 months differed in some unmeasured factors or motivations that may have impacted the results, including impacts of the pandemic.

## Conclusion

This study highlights that scalable interventions for young adolescents in LMICs may need to consider structures that offer ongoing support to optimise the likelihood of treatment strategies being provided in the programmes being used in the long-term. These strategies may need to consider the changing contextual stressors experienced by adolescents and their caregivers in LMICs, and longer-term strategies offered may need to be flexibly administered to match the potentially changing needs of people. It is also important to note that the distinct contextual factors that young adolescent Syrian refugees face in Jordan, including uncertainty about their future and challenging economic prospects, can modulate the long-term effects of EASE. Further, social media and peer influence may interact with the strategies promoted in EASE, and these may influence the extent to which the strategies are rehearsed in the long-term after participation in EASE is completed. If optimal mental health is to be achieved among young adolescents in LMICS, it is important that long-term effects are considered and evaluated.

## Supporting information

Bryant et al. supplementary material Bryant et al. supplementary material

## Data Availability

The data that support the findings of this study are publicly available at the time of publication of this manuscript. The full trial data can be requested from the corresponding author with reasonable requests.

## Supplementary material

The supplementary material for this article can be found at https://doi.org/10.1017/S2045796024000817
